# Lenalidomide in the treatment of anti‐myelin‐associated glycoprotein neuropathy: A phase 1 study to identify the maximum tolerated dose

**DOI:** 10.1111/ene.16164

**Published:** 2023-11-28

**Authors:** Amro M. Stino, Naresh Bumma, Rachel Smith, Long Davalos, Jeff Allen, Jing Christine Ye, Matthew Pianko, Erica Campagnaro, Cassandra Fierro, Abdelrahman Awad, Ben Murdock, Maciej Pietrzak, Gerard Loszanski, David M. Kline, Yvonne Efebera, Bakri Elsheikh

**Affiliations:** ^1^ Department of Neurology, Division of Neuromuscular Medicine The University of Michigan Health System Ann Arbor Michigan USA; ^2^ Department of Internal Medicine, Division of Hematology‐Oncology The Ohio State University Wexner Medical Center Columbus Ohio USA; ^3^ Center for Biostatistics The Ohio State University Wexner Medical Center Columbus Ohio USA; ^4^ Department of Neurology, Division of Neuromuscular Medicine The University of Cincinnati Medical Center Cincinnati Ohio USA; ^5^ Department of Neurology, Division of Neuromuscular Medicine University of Minnesota Minneapolis Minnesota USA; ^6^ Department of Internal Medicine, Division of Hematology‐Oncology The University of Michigan Health System Ann Arbor Michigan USA; ^7^ Department of Biomedical Informatics The Ohio State University Wexner Medical Center Columbus Ohio USA; ^8^ Department of Pathology The Ohio State University Wexner Medical Center Columbus Ohio USA; ^9^ Department of Biostatistics and Data Science, Division of Public Health Sciences Wake Forest University School of Medicine Winston‐Salem North Carolina USA; ^10^ Department of Oncology Ohio Health Columbus Ohio USA; ^11^ Department of Neurology, Division of Neuromuscular Medicine The Ohio State University Wexner Medical Center Columbus Ohio USA

**Keywords:** anti‐MAG neuropathy, drug safety, drug trial, lenalidomide, outcome measures

## Abstract

**Background:**

Anti‐myelin‐associated glycoprotein (MAG) neuropathy is a debilitating demyelinating polyneuropathy with no approved therapies. Our primary objective was to ascertain lenalidomide safety and maximum tolerated dose (MTD) in anti‐MAG neuropathy.

**Methods:**

This phase 1b, open‐label, single‐arm, dose‐finding trial was conducted from 2019 through 2022. The original design included a dose‐escalation/extension phase followed by a dose‐expansion phase. Three doses of lenalidomide were evaluated: 10, 15, and 25 mg. The main outcome was the MTD.

**Results:**

Eleven patients enrolled (10 men), with a mean age of 67.6 years (SD = 6.18, range 58–77 years) and mean disease duration of 8.5 years (SD = 10.9, range 1–40 years). The study terminated early due to higher‐than‐expected non‐dose‐limiting toxicity venous thromboembolism (VTE) events. The calculated MTD was 25 mg (posterior mean of toxicity probability was 0.01 with a 95% credible interval of 0.00, 0.06), but a recommended phase 2 dose of 15 mg was advised. For secondary exploratory outcomes, only EQ‐5D (−0.95, 95% CI −1.81 to −0.09) and total IgM (−162 mg/dL, 95% CI −298 to −26) showed signs of improvement by month 12.

**Conclusions:**

Lenalidomide was associated with higher‐than‐expected VTE events in anti‐MAG neuropathy patients, despite a calculated MTD of 25 mg. A recommended phase 2 dose of 15 mg was advised. Lenalidomide did not improve disability or impairment at 12 months, although this study was not powered for efficacy. The risks of long term lenalidomide may outweigh benefit for patients with anti‐MAG neuropathy. Any future efficacy study should address VTE risk, as current myeloma guidelines appear inadequate.

**Trial Registration:**

Lenalidomide in Anti‐MAG Neuropathy: Phase 1b Study, ClinicalTrials.gov Identifier: NCT03701711, https://clinicaltrials.gov/ct2/show/NCT03701711. First submitted October 10, 2018. First patient enrolled in January 2019.

## INTRODUCTION

Anti‐myelin‐associated glycoprotein (MAG) neuropathy is a large fiber, sensory‐predominant, demyelinating polyneuropathy with a prevalence of 1 per 100,000, and a male : female prevalence of nearly 3 : 1 [1, 2, 3]. Pathogenic IgM monoclonal antibodies target the MAG protein, which is key for myelin sheath formation and stability [4, 5, 6]. The neuropathy is characterized clinically by distal sensory loss, gait imbalance, ataxia, and distal weakness, all contributing to long‐term disability [7]. No proven effective or US Food and Drug Administration (FDA)‐approved therapies exist for anti‐MAG neuropathy [8, 9]. Of all studied therapies, rituximab has gained the most enthusiasm. Although uncontrolled studies suggest that 30%–50% of patients benefit from rituximab treatment, two randomized controlled clinical trials failed to meet primary endpoints [10, 11, 12].

Lenalidomide (Revlimid; Celgene Corporation, Summit, NJ, USA), a thalidomide analogue, is an immunomodulatory agent that inhibits pro‐inflammatory cytokines and increases anti‐inflammatory cytokines from peripheral blood mononuclear cells [13]. Lenalidomide, in combination with dexamethasone, is approved for newly diagnosed multiple myeloma [14] and shows efficacy in other plasma cell dyscrasias, such as amyloidosis [15] and polyneuropathy, organomegaly, endocrinopathy, monoclonal gammopathy, and skin changes (POEMS) [14, 16]. Its efficacy in POEMS polyneuropathy is striking, with improvement in 92% of patients and stabilization in the remaining 8% [17]. In addition, lenalidomide carries a favorable safety profile relative to thalidomide [17]. Lenalidomide at doses of 5–25 mg has shown benefit in individual cases of anti‐MAG neuropathy [18, 19]. Lenalidomide has been shown to inhibit IgM antibody synthesis both in vitro [20] and in IgM multiple myeloma [21], and we have observed marked clinical improvements in rituximab‐refractory anti‐MAG neuropathy patients treated with lenalidomide. For these reasons we aimed to assess the safety and optimal dose of lenalidomide in anti‐MAG neuropathy as well as to explore therapeutic efficacy. We hypothesized that the maximum tolerated dose (MTD) would be 25 mg, given the good tolerability of the drug in clinical practice.

## METHODS

### Overview

This phase 1b, open‐label, single‐arm, non‐randomized, dose‐finding safety study aimed to evaluate the MTD of lenalidomide in anti‐MAG neuropathy. As a dose‐finding study, this trial was not powered to conclusively ascertain drug efficacy, but instead to select the MTD, assess safety, and explore therapeutic efficacy using a broad range of outcome measures. Enrollment occurred from January 2019 through February 2022 at The Ohio State University Wexner Medical Center and The University of Michigan. The original study design was to have a dose‐escalation/dose‐extension phase (1–2 years), followed by a dose‐expansion (1 year) phase.

### Standard protocol approvals, registrations, and patient consents

Institutional Review Board (IRB) approval was obtained at both The Ohio State University Wexner Medical Center Biomedical Sciences IRB (00000294) and The University of Michigan Medical Campus IRB (IORG0000144). This study was performed with the full understanding and written informed consent of all patients and conforms with the World Medical Association Declaration of Helsinki. The study was registered with ClinicalTrials.gov (NCT03701711).

### Patient selection

Patients 18 years of age or older with a diagnosis of anti‐MAG neuropathy were eligible to participate. Patients with Waldenström's macroglobulinemia or other plasma cell malignancies not receiving systemic chemotherapy, as deemed per hematology evaluation, were eligible. All enrollees were required to have an IgM monoclonal protein spike, an elevated anti‐MAG titer of at least 6000 as measured via enzyme‐linked immunosorbent assay (ELISA) (Buhlmann, Schönenbuch, Switzerland), with results expressed as Buhlmann titer units (BTU), and electrodiagnostic evidence of a demyelinating polyneuropathy, as codified in the European Academy of Neurology/Peripheral Nerve Society (EAN/PNS) criteria [22]. Nerve conduction studies were performed at baseline to ensure fulfillment of demyelinating criteria. The right fibular, tibial, median, and ulnar motor nerves and the right sural, median, and ulnar sensory nerves were assessed in all patients. Patients with renal failure (serum creatinine ≥2 mg/dL or calculated creatinine clearance ≤40 mL/min) or hepatic failure (total bilirubin ≥1.5 mg/dL, alkaline phosphatase ≥3X, and AST/ALT ≥2X the institutional upper limit of normal), thrombocytopenia (platelets <75,000/μL), absolute neutropenia (<1000/μL), or those actively receiving systemic chemotherapy were excluded. Use of lenalidomide or any other immunosuppressive therapy (including rituximab) in the preceding 6 months was not permitted. However, intravenous immunoglobulin (IVIG) was allowed if patients were at a stable dose and frequency in the 6 months leading up to enrollment.

### Study design

The study was designed to evaluate the safety profile of three doses of lenalidomide: 10, 15, or 25 mg, administered in an open‐label fashion. A 2015 phase 1/2 study assessing lenalidomide in Waldenström macroglobulinemia patients that evaluated doses of 15, 20, and 25 mg recommended a dose of 15 mg, due to neutropenic sepsis and fatigue at 20 mg [23]. Furthermore, clinically significant anemia occurred at doses of 25 mg or higher in previous studies. Our decision to study 10, 15, or 25 mg stemmed from previous case reports in anti‐MAG neuropathy patients showing a favorable safety profile at doses ranging from 5 to 25 mg [18, 19]. In addition, anecdotal experience from our anti‐MAG neuropathy clinic patients receiving off‐label lenalidomide at doses as high as 20–25 mg showed no venous thromboembolism (VTE), anemia, neutropenia, or fatigue toxicity concern.

Drug was shipped directly from the manufacturer to each patient through a lenalidomide Risk Evaluation and Mitigation Strategy (REMS) program. Patients were provided with a pill diary. Drug was self‐administered by each patient at home on days 1–21 of every 28‐day cycle and taken in conjunction with dexamethasone 20 mg (days 1, 8, 15, and 22 of each 28‐day cycle). To minimize the VTE risk of lenalidomide, patients were assigned prophylaxis per the supervising hematologist and in accordance with VTE myeloma guidelines. Depending on the risk, patients received 81–325 mg aspirin daily, full dose warfarin (target international normalized ratio [INR] 2–3), 2.5 mg or greater of apixaban twice daily, low molecular weight heparin, or 10–20 mg rivaroxaban daily.

The original study design contained two components. The dose‐escalation/dose‐extension phase aimed to recruit 12 patients for 1–2 years to ascertain MTD. The follow‐up dose‐expansion phase would then enroll a separate eight patients to receive the ascertained MTD for up to 1 year. Thus, the original aim was for a total enrollment of 20 patients. Safety and efficacy assessments were conducted with each cycle for the first three cycles, followed by assessments every three cycles until the end of year 1. For patients enrolled up to 2 years, assessments in year 2 were done every six cycles.

### Scoring of primary safety outcome

To ascertain dose‐limiting toxicity (DLT), periodic blood draws and safety screening questionnaires were obtained. Blood testing included a complete blood count with differential and a comprehensive metabolic panel. Self‐reported safety monitoring included a screening checklist specifically evaluating for DLT events and the lenalidomide REMS screening, required by the drug manufacturer. DLT was defined using a prespecified grading scale (Table S1). To qualify as a DLT, the event had to occur during cycle 1 of drug therapy. Any event that occurred thereafter (cycle 2 onwards) was deemed a non‐DLT adverse event (be it serious or non‐serious) and did not affect the MTD calculation.

### Scoring of exploratory efficacy outcomes

Secondary exploratory efficacy outcome measures included change in disability as assessed by the Overall Neuropathy Limitations Scale (ONLS) and the Inflammatory Rasch‐built Overall Disability Scale (I‐RODS) as well as change in quality of life as assessed by the European Quality—5D‐5L questionnaire. Physical examination metrics included the Scale for the Assessment and Rating of Ataxia (SARA), the Muscle Research Council Summated Score (MRCSS) (scored from 0 to 60), and Jamar grip strength testing of both hands (using best of two trials per limb). Fatigue was assessed by the Fatigue Severity Scale (FSS). Biomarker measures consisted of anti‐MAG titer level, IgM (monoclonal) level, and IgM (total) level. Flow cytometry was performed on three patients from The Ohio State University and involved baseline and cycle 12 comparisons for the following markers, which were based on a second selection of markers deemed clinically relevant: CD4+ T cells (CD3+ CD4+ CD8−), CD8+ T cells (CD3+ CD4− CD8+), B cells (CD3− CD19+), NK cells (CD3− CD56^mid^ CD16+, NK (CD56+) cells, T‐reg cells (CD3+ CD4+ CD25+ CD127−), and monocytes (CD14+). Immunome data were preprocessed and visualized using R programming language (R Core Team, 2022. R: A language and environment for statistical computing. R Foundation for Statistical Computing, Vienna, Austria, https://www.R‐project.org/).

### Statistical analysis

The starting dose of lenalidomide was assigned at the time of patient enrollment and driven by the occurrence of DLT events in preceding enrollments. To find the MTD (primary outcome) and select the dose level for each cohort enrolled, a Bayesian Optimal Interval Design (BOIN) was used [24]. The target toxicity rate was set at 0.3 and the maximum sample size at 12 patients. We aimed to enroll in cohorts of size 1 but with the flexibility to modify subsequent cohort sizes as desired. After the enrollment of the maximum sample size, the MTD was to be selected using isotonic regression. The MTD was designated as the dose with the estimated toxicity rate closest to the target rate of 0.3. Patients who had not progressed and who experienced unacceptable toxicity were eligible for re‐treatment at a lower dose. A maximum of two dose reductions were to be allowed prior to withdrawal. Calculations were done using the BOIN package in R. Exploratory evaluation of therapeutic efficacy estimates (secondary outcome measures) was conducted using linear mixed models with a categorical effect of time and random intercepts to assess change over time and account for loss to follow‐up. Models were fit using SAS 9.3 (SAS Institute Inc., Cary, NC, USA). Contrasts comparing cycle 12 and baseline were extracted from the model for each outcome.

## RESULTS

### Patient baseline characteristics

**FIGURE 1:**
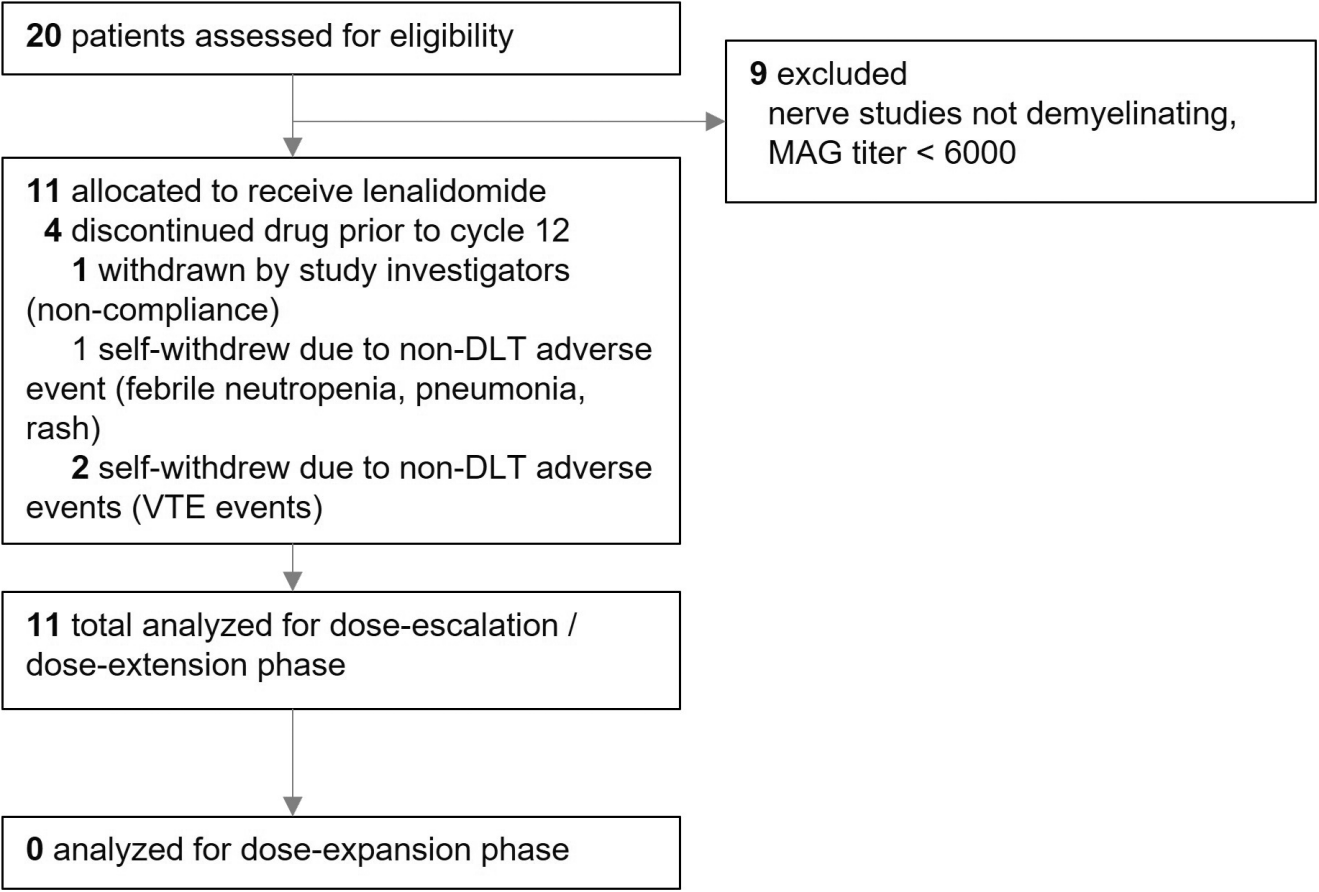
Study flow diagram. DLT, dose‐limiting toxicity; MAG, myelin‐associated glycoprotein; VTE, venous thromboembolism.

We screened a total of 20 patients and ultimately enrolled 11 in our dose‐escalation/dose‐extension phase (Figure 1). Three patients were excluded due to low MAG titers and six due to lack of demyelinating findings on nerve conduction studies. No patients were enrolled in the dose‐expansion phase due to early study termination due to safety concerns. The enrolled dose‐escalation/extension cohort consisted of 10 men and 1 woman, with a mean age of 67.6 years (SD = 6.18, range 58–77 years) and mean disease duration of 8.5 years (SD = 10.9 years, range 1–40 years) (Table 1). The study lasted from January 2019 through February 2022 (last study visit).

**TABLE 1:** Patient baseline characteristics.

Characteristic	Value (*N* = 11)
Demographics	
Male	10 (91%)
Age (mean), years (SD)	67.6 (6.18)
Disease duration (mean), years (SD)	8.5 (10.9)
Baseline hematologic disease	
MGUS only	9 (82%)
Waldenström macroglobulinemia	1 (9%)
CLL	1 (9%)
ITP	1 (9%)
Previous treatments	
IVIG	4 (36%)
Rituximab	4 (36%)
Cyclophosphamide	1 (9%)
Baseline clinical disease activity	
ONLS (mean), units	3.18
IRODS percentile (mean)	75.58
FSS (mean), units	33.18
EQ‐5D (mean), units	4.27
SARA (mean), units	10
MRCSS (mean), units	57.45
Baseline biomarker levels	
Patients with MAG titer ≥102,000 BTU	9 (82%)
IgM monoclonal (mean), mg/dL	276.6
IgM total (mean), mg/dL	555.82
MYD88^L265P^ mutational variant (positive/tested)	1/4 (25%)
CXCR4^S338X^ mutational variant (positive/tested)	0/4 (0%)
Baseline hepatic and renal function	
Total bilirubin (mean), mg/dL	0.7
Alkaline phosphatase (mean), IU/L	85
AST (mean), IU/L	24
ALT (mean), IU/L	25
BUN (mean), mg/dL	16.90
Creatinine (mean), mg/dL	0.96

Abbreviations: ALT, alanine aminotransferase; AST, aspartate aminotransferase; BTU, Buhlmann titer units; BUN, blood urea nitrogen; CLL, chronic lymphocytic leukemia; EQ‐5D, EuroQol‐5 Dimension; FSS, Fatigue Severity Scale; IRODS, Inflammatory Rasch‐built Overall Disability Scale; ITP, immune thrombocytopenic purpura; IVIG, intravenous immunoglobulin; IU, international unit; MAG, myelin‐associated glycoprotein; MGUS, monoclonal gammopathy of unknown significance; MRCSS, Medical Research Council Summated Score; ONLS, Overall Neuropathy Limitations Scale; SARA, Scale for the Assessment and Rating of Ataxia; SD, standard deviation.

### Safety and MTD

Due to a higher‐than‐anticipated occurrence of non‐DLT VTE events (three pulmonary embolus [PE] events in three separate patients, with two concomitant deep vein thrombosis [DVT] events in two of those patients), the study terminated early, and the dose‐expansion phase was not pursued. Details regarding VTE events are summarized in Table 2. No formal DLT events occurred. The final study MTD was 25 mg based on dose‐finding analysis from the 11 patients who completed at least one cycle of the dose‐escalation/extension phase. The posterior mean of toxicity probability was 0.01 with a 95% credible interval of 0.00, 0.06. The posterior probability that toxicity probability was greater than the target toxicity probability of 0.3 was 0.04. Despite the calculated MTD being 25 mg, a recommended phase 2 dose (RP2D) of 15 mg was chosen by study investigators. Our decision to dose‐reduce to 15 mg was made following the second of three PE events. Actively enrolled patients taking 25 mg were also switched to 15 mg for the remainder of the enrollment period. A more aggressive VTE prophylactic regimen with low‐dose direct oral anticoagulation was instituted for all new enrollees, given the absence of clear guidelines on optimal VTE risk reduction, and the persistently high VTE occurrence rate. With occurrence of the third PE event, it was decided to terminate the study, thus precluding the formal dose‐expansion phase. Of note, all three VTE events occurred in patients who were risk‐stratified to receive antiplatelet therapy per the original myeloma guidelines, with none occurring in those patients on anticoagulants.

**TABLE 2:** Summary of venous thromboembolism events.

Patient	Drug cycle at VTE event	Age (years), sex	Baseline VTE risk factors + CCS dose	Entry MAG titer (BTU)	Entry IgM (spike) level, (g/dL)	Entry ONLS	PE (CT) or DVT (US) imaging findings	Study drug dose at time of VTE	VTE prophylaxis at time of VTE event	Post‐VTE event study drug dose
#5	6	72, M	A‐Fib, previous PE, CKD Weekly Dexa 20 mg	Titer not obtained	244	6	CT: R lower lobe occlusive PE with developing R lower lobe pulmonary infarct, with cardiomegaly and pulmonary arterial hypertension US: none	25 mg	ASA 325 mg daily	Stopped study drug but stayed in study through cycle 9
#7	3	68, M	None Weekly Dexa 20 mg	102,400	Level not obtained	2	CT: saddle pulmonary embolism with thrombus extending into bilateral distal main and proximal lobar branches US: acute DVT L leg in popliteal, tibial, and peroneal veins	25 mg	ASA 81 mg daily	15 mg
#8	9	66, M	CAD Weekly Dexa 20 mg	102,400	525	1	CT: nonobstructive segmental PE in R lower lobe and obstructive subsegmental PE in L lower lobe with infarct US: acute DVT in R peroneal vein and L peroneal and posterior tibial veins	15 mg	ASA 81 mg daily	Stopped study drug

Abbreviations: A‐Fib, atrial fibrillation; ASA, aspirin; BTU, Buhlmann titer units; CAD, coronary artery disease; CKD, chronic kidney disease; CT, computed tomography; Dexa, dexamethasone; DVT, deep vein thrombosis; L, left; M, male; MAG, myelin‐associated glycoprotein; ONLS, Overall Neuropathy Limitations Scale; PE, pulmonary embolus; R, right; US, ultrasound; VTE, venous thromboembolism.

In addition to the VTE events, one patient experienced two non‐DLT serious adverse events during cycle 2 (febrile neutropenia with pneumonia and skin rash), for which he was hospitalized, and for which the patient self‐withdrew from the study. Notably, this patient had baseline chronic lymphocytic leukemia. One additional patient was voluntarily withdrawn by study investigators at cycle 3 due to drug non‐compliance. Of the three patients who experienced VTE events, two self‐withdrew in cycle 9 (patients #5 and #8). Patient #5 experienced a VTE event (PE only) in cycle 6 but remained enrolled in study (off study drug) through cycle 9, at which point he disenrolled. Patient #8 experienced his VTE events (PE and DVT) in cycle 9 and exhibited study drug non‐responsiveness, prompting his disenrollment. All in all, 4 of the 11 patients did not complete the planned minimum 1‐year period for the dose escalation/extension phase of the study. DLTs, adverse events, and serious adverse events are summarized in Table 3.

**TABLE 3:** Adverse events.

Adverse event	Patients (*n* (%)) (*N* = 11)	Cycle #
VTE (DVT)	2 (18)	#3 (2 events), #9 (1 event)
VTE (PE)	3 (27)	#3 (2 events), #6 (1 event), #9 (1 event)
Febrile neutropenia	1 (9)	#2
Pneumonia	1 (9)	#2
Skin rash	1 (9)	#2

*Note*: No formal DLT events occurred (defined as occurring in cycle 1).

Abbreviations: DVT, deep vein thrombosis; PE, pulmonary embolus; VTE, venous thromboembolism.

### Exploratory efficacy outcomes

**FIGURE 2:**
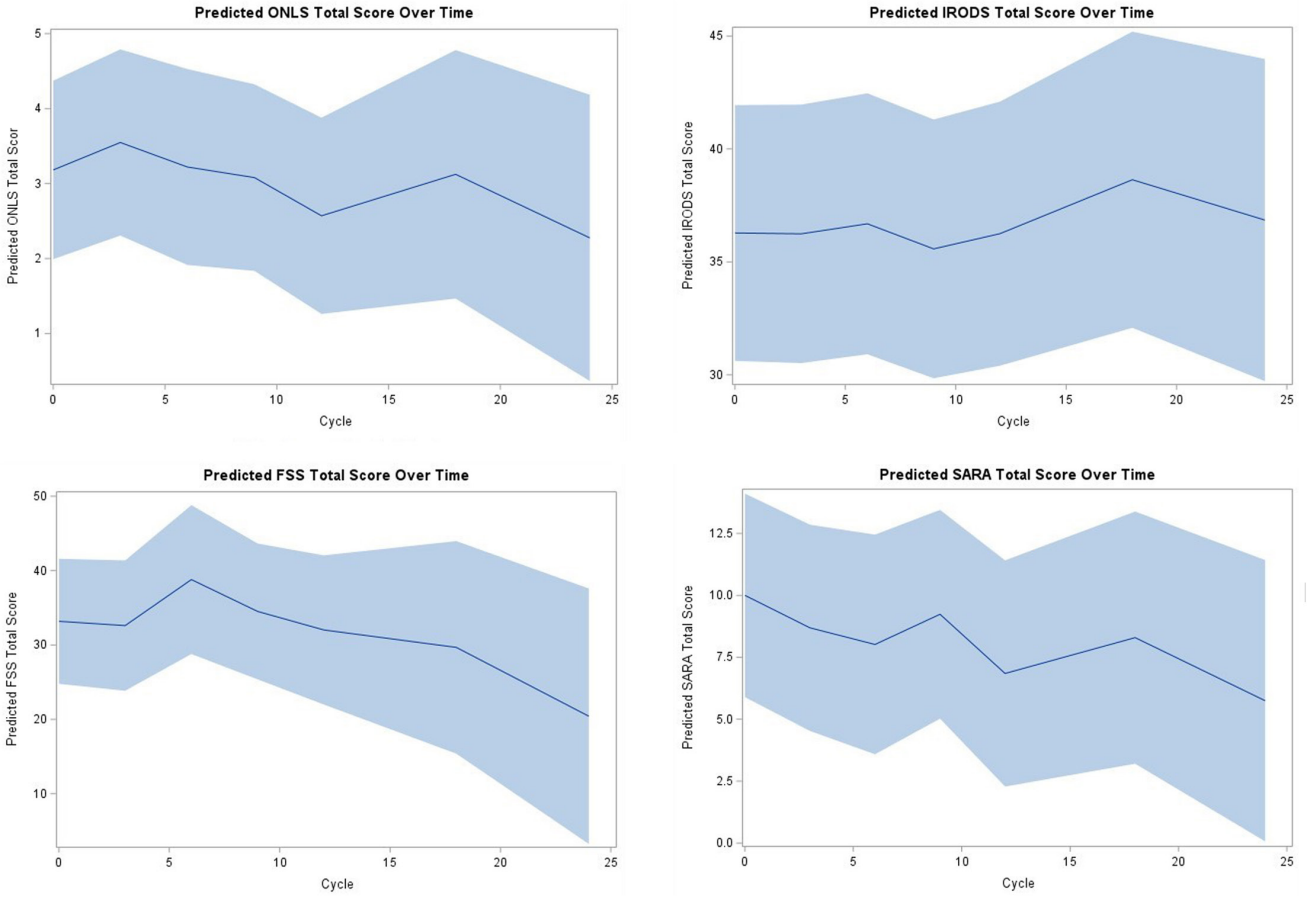
Predicted mean values across time for clinicometric efficacy outcome measures. None of the efficacy outcome measures met statistical significance, but there was a trend towards improvement in the ONLS, FSS, and SARA. FSS, Fatigue Severity Scale; IRODS, Inflammatory Rasch‐built Overall Disability Scale; ONLS, Overall Neuropathy Limitations Scale; SARA, Scale for the Assessment and Rating of Ataxia.

The results of predicted mean outcome, conducted using linear mixed models with random intercepts, are presented in Figure 2 and Table 4, with source data available in Table S2. At 1 year, 7 of 11 patients remained on treatment and signs of improvement were seen for EQ‐5D (−0.95, 95% CI −1.81 to −0.09) and IgM (total) (−162 mg/dL, 95% CI −298 to −26), albeit not IgM (monoclonal). Neither SARA (−3.15, 95% CI −6.49 to 0.18) nor other efficacy measures, namely ONLS (total), ONLS (leg), IRODS, FSS, Jamar, or MRCSS, were significant. No patterns emerged that correlated flow cytometry to clinical changes (Figure S1).

**TABLE 4:** Estimated means and 95% confidence intervals for selected outcomes over the study period.

Measure	Baseline *(N* = 11)	Cycle 3 *(N* = 11)	Cycle 6 *(N* = 8)	Cycle 9 *(N* = 9)	Cycle 12 *(N* = 7)	Cycle 18 *(N* = 3)	Cycle 24 *(N* = 2)
ONLS‐total, units	3.18 (1.99, 4.37)	3.55 (2.31, 4.79)	3.22 (1.91, 4.53)	3.08 (1.84, 4.32)	2.57 (1.26, 3.88)	3.12 (1.47, 4.78)	2.28 (0.37, 4.18)
ONLS‐leg, units	1.91 (1.04, 2.78)	2.22 (1.33, 3.11)	1.84 (0.93, 2.76)	2.11 (1.22, 3.01)	1.6 (0.68, 2.51)	1.97 (0.92, 3.02)	0.97 (−0.19, 2.12)
IRODS, percentile	75.58 (63.79, 87.38)	75.50 (63.58, 87.42)	76.43 (64.39, 88.46)	74.12 (62.18, 86.05)	75.52 (63.34, 87.70)	80.49 (66.84, 94.15)	76.78 (61.92, 97.63)
FSS total, units	33.18 (24.77, 41.59)	32.61 (23.84, 41.37)	38.79 (28.78, 48.80)	34.51 (25.37, 43.64)	32.02 (21.99, 42.05)	29.67 (15.38, 43.97)	20.42 (3.25, 37.60)
EQ‐5D, units	4.27 (3.33, 5.21)	4.61 (3.65, 5.58)	3.67 (2.67, 4.67)	3.89 (2.91, 4.87)	3.32 (2.29, 4.35)	3.01 (1.72, 4.31)	3.88 (2.39, 5.38)
SARA, units	10 (5.89, 14.11)	8.69 (4.53, 12.85)	8.02 (3.58, 12.45)	9.24 (5.02, 13.45)	6.85 (2.28, 11.41)	8.29 (3.2, 13.38)	5.75 (0.07, 11.42)
Jamar right, lbs	61.14 (40.44, 81.83)	64.6 (43.86, 85.34)	61.14 (40.19, 82.1)	58.73 (37.94, 79.51)	60.3 (39.41, 81.18)	55.95 (34.43, 77.47)	55.38 (33.3, 77.47)
Jamar left, lbs	56.41 (38.67, 74.14)	63.6 (45.8, 81.4)	61.75 (43.61, 79.88)	56.3 (38.43, 74.17)	62.85 (44.83, 80.88)	64.05 (45.04, 83.07)	53.31 (33.42, 73.2)
MRCSS, units	57.45 (55.54, 59.37)	57.91 (55.99, 59.83)	57.44 (55.44, 59.44)	57.71 (55.77, 59.65)	58.02 (55.98, 60.05)	58.46 (56.28, 60.64)	57.26 (54.91, 59.61)
IgM monoclonal (mg/dL)	276.6 (89.9, 463.2)	–	–	–	173.37 (−18.97, 365.72)	–	–
IgM total (mg/dL)	555.82 (288.60, 823.03)	–	–	–	393.8 (123.16, 664.50)	–	–

Abbreviations: BTU, Buhlmann titer units; EQ‐5D, EuroQol‐5 Dimension; FSS, Fatigue Severity Scale; IRODS, Inflammatory Rasch‐built Overall Disability Scale; MAG, myelin‐associated glycoprotein; MRCSS, Medical Research Council Summated Score; ONLS, Overall Neuropathy Limitations Scale; SARA, Scale for the Assessment and Rating of Ataxia.

## DISCUSSION

This phase 1b, dose‐finding study of lenalidomide in patients with anti‐MAG neuropathy showed a higher‐than‐anticipated VTE occurrence resulting in early study termination. Despite no formal DLT events and a calculated MTD of 25 mg, a recommended phase 2 dose of 15 mg was instituted due to VTE concern. While exploratory efficacy data were collected, findings were limited by the phase 1 nature of the study (as a safety study), early study termination, and limited patient recruitment.

Our study provides valuable short‐ and long‐term (up to 2 years) data on VTE and other safety risk in anti‐MAG patients receiving lenalidomide. The frequency of VTE events in our study is higher than other lenalidomide studies, and the effect of lenalidomide dose on VTE risk remains unclear. Data from multiple myeloma studies have shown a reduced VTE risk with 15 mg dosing [25], especially in patients >60 or 75 years of age [26, 27], without reduction in therapeutic efficacy, although the dose‐related nature of lenalidomide‐related VTE toxicity remains controversial. In general, previous lenalidomide studies in multiple myeloma show DVT and PE frequencies ranging from 4%–11.9% to 3.4%–4.4%, respectively [28, 29], which are reduced to <1% with a risk‐adapted approach to prophylaxis [30], and an overall VTE incidence of 7% in primary or light chain amyloidosis (AL) [31, 32]. In POEMS syndrome, one open‐label study of 15 patients showed no VTE occurrence after six cycles for patients receiving combined lenalidomide 25 mg and 40 mg of weekly dexamethasone therapy [33]. Patients in that study were placed on either 100 mg daily aspirin for VTE prophylaxis, or low molecular weight heparin (if aspirin‐intolerant). In general, VTE risk in lenalidomide‐treated patients increases with high‐dose glucocorticoids (dexamethasone 40 mg/day by mouth on days 1–4, 9–12, and 17–20 of each 28‐day cycle), as compared to low‐dose glucocorticoids (dexamethasone 40 mg/day on days 1, 8, 15, and 22 of each 28‐day cycle). With regards to non‐VTE‐related dose tolerability, lenalidomide had an MTD of 15 mg in a phase 1/2 dose‐escalation trial in AL amyloidosis [34], and was better tolerated than 25 mg [15]. In addition, the previously discussed 2015 phase 1/2 study exploring lenalidomide usage in Waldenström's macroglobulinemia recommended a dose of 15 mg (rather than 20 or 25 mg) due to neutropenic sepsis, fatigue, and anemia, none of which were concerns in our study [23]. None of the 17 subjects in the 2015 study experienced VTE events, despite a VTE prophylaxis algorithm identical to ours, thus raising the question of whether the high VTE risk in our study was a disease‐specific concern unique to anti‐MAG neuropathy patients.

The reasons for the higher‐than‐expected occurrence of VTE in our study is unknown. Potential factors may include increased age of study patients (mean age 67.6 years), although multiple myeloma patients are typically of similar age. Reduced mobility due to the SARS‐CoV‐2 pandemic is a possible cause, although no published data substantiate this in the myeloma literature [35]. Furthermore, none of our patients contracted SARS‐CoV‐2. One cannot implicate immobility alone, for POEMS syndrome patients often have comparable immobility to patients with anti‐MAG neuropathy, but do not have such a high VTE occurrence with lenalidomide. In addition, there did not appear to be a clear association between IgM levels or MAG titers and VTE risk in our study, although the small size of our study limited our ability to fully investigate this potential association. Interleukin‐6 (IL‐6) could be a target of future research with regards to VTE risk in patients with anti‐MAG neuropathy, as they have higher median IL‐6 levels than healthy controls [36].

Adequate VTE prophylaxis in the context of lenalidomide and anti‐MAG neuropathy is a challenging topic. Multiple myeloma VTE prophylaxis algorithms are driven by steroid or concurrent chemotherapy use. Aspirin monotherapy is recommended for patients not receiving steroids, those not receiving combination chemotherapy, and those receiving steroids and one chemotherapy agent (and having less than one VTE risk factor) [37]. For patients receiving high‐dose corticosteroids, doxorubicin, multiagent chemotherapy, or having more than one risk factor, anticoagulation is advised. Stated risk factors include immobilization and body mass index >30 kg/m^2^, but not age. Our study raises the important question of whether the multiple myeloma VTE risk stratification algorithm is adequate for anti‐MAG neuropathy.

Although our study was not powered to demonstrate efficacy, we did find signs of improvement in EQ‐5D at month 12 compared to baseline. In addition, there was a decline in IgM (total) level. While SARA showed the greatest improvement, neither SARA nor other efficacy outcome measures, including ONLS, were significantly different at 1 year. While IgM (total) levels consistently declined, neither IgM (monoclonal) nor MAG titers did, nor did they consistently reflect therapeutic efficacy in ONLS‐responders.

Current anti‐MAG neuropathy clinical trials are limited by outcome measures insensitive at capturing therapeutic efficacy. In the placebo arms of the 2009 and 2013 rituximab clinical trials [38], Inflammatory Neuropathy Cause and Treatment (INCAT) disability deteriorated by just 0.09 points at 8 months in one study and showed no change at 12 months in the other study [11]. The slow natural history of the disease also makes it challenging to demonstrate efficacy, especially if improvement of long‐standing axonal nerve injury is not feasible and existing outcome measures are insufficiently sensitive to differentiate stability from slow deterioration at a relatively short interval of 8 or 12 months. We elected to use the ONLS disability scale as the primary determinant of efficacy instead of the INCAT disability scale. Unlike the INCAT scale, the ONLS scale incorporates activities like climbing stairs and running into lower limb scoring. We hypothesized that this would give the score a higher ceiling, and potentially better capture such subtle but important changes in anti‐MAG neuropathy [39]. Although four of our patients showed improvements in ONLS, the remaining seven were stable or worse at the final study assessment.

With regards to biomarkers, a recent meta‐analysis suggests that MAG titer correlates with clinical response, with a 50% drop suggesting a favorable response [40]. In the study, non‐responders showed a minimal decline in MAG titer (only 11%), while those who acutely deteriorated showed a 204% increase in titer [41]. Others, however, have questioned the utility of MAG titer in monitoring disease activity [42]. Like the 2009 rituximab trial, we found a decline in mean IgM (total) level from baseline to post‐treatment, although the specificity of IgM (total or monoclonal) as an outcome measure is unknown. In terms of flow cytometry in our study, lenalidomide suppressed B cell levels, while monocyte counts increased. The 2009 trial showed increase in CD25 + CD4 + Foxp3+ regulatory cells by month 8 after treatment and a slight increase in ICOS+ cells by month 6 [10]. However, no other flow cytometry signal was seen. Overall, there was no consistent association between clinicometric and biomarker outcome measures as indicators of therapeutic efficacy in our study. The one patient who demonstrated decline in B cell count on flow cytometry (and was a clinical responder per ONLS) showed no decline in MAG titer, a finding which questions the utility of MAG titer as a consistent effector function biomarker.

Our study had certain limitations, namely the small sample size, the open‐label nature of the study, as well as lack of a dose‐expansion phase, due to early termination from VTE occurrence. The lack of validated outcome measures for anti‐MAG neuropathy further limits our ability to draw definitive conclusions on drug efficacy.

## CONCLUSIONS

Patients with anti‐MAG neuropathy receiving lenalidomide therapy appear to be at higher‐than‐anticipated risk for developing VTE events. Existing multiple myeloma VTE risk algorithms are inadequate for patients with anti‐MAG neuropathy. If lenalidomide is administered, strong consideration for anticoagulation therapy and co‐management with hematology is encouraged. As therapeutic efficacy was an exploratory objective, we did not observe any consistent clinical or biologic signal of treatment benefit. Taken together, adequate VTE prophylaxis in this patient population warrants particular attention should lenalidomide carry any potential future viability as a therapeutic consideration for anti‐MAG neuropathy.

## AUTHOR CONTRIBUTIONS

**Amro M. Stino:** Conceptualization; investigation; funding acquisition; writing – original draft; methodology; writing – review and editing; formal analysis; data curation; resources; project administration; supervision. **Naresh Bumma:** Investigation; methodology; formal analysis; data curation. **Rachel Smith:** Methodology; validation; formal analysis; data curation. **Long Davalos:** Data curation. **Jeff Allen:** Writing – review and editing; methodology; formal analysis; supervision. **Jing Christine Ye:** Data curation; methodology; investigation. **Matthew Pianko:** Methodology; data curation; investigation. **Erica Campagnaro:** Methodology; data curation; investigation. **Cassandra Fierro:** Data curation. **Abdelrahman Awad:** Data curation. **Ben Murdock:** Formal analysis; methodology; writing – review and editing; software. **Maciej Pietrzak:** Writing – review and editing; data curation; methodology; formal analysis; software. **Gerard Loszanski:** Software; formal analysis; supervision; methodology. **David M. Kline:** Conceptualization; investigation; writing – review and editing; methodology; formal analysis; supervision. **Yvonne Efebera:** Conceptualization; investigation; writing – review and editing; methodology; formal analysis; supervision. **Bakri Elsheikh:** Investigation; methodology; writing – review and editing; formal analysis; supervision; data curation.

## FUNDING INFORMATION

This study was supported through a GBS‐CIDP Foundation Discovery Award (University of Michigan) as well as The Ohio State Myeloma Fund (The Ohio State University). Study drug was made available through Celgene (Bristol Myers Squibb).

## CONFLICT OF INTEREST STATEMENT

Amro M. Stino has served as a consultant for Argenx and CSL Behring. He has received research funding from the GBS‐CIDP Foundation and BMS/Celgene. Naresh Bumma has served as a consultant and on speaker's bureaus for Amgen, Sanofi, and Janssen. Jeff Allen has served as a consultant for Alexion, Alnylym, Argenx, Akcea, Annexon, CSL Behring, Johnson & Johnson, Grifols, Takeda, Immunovant, Immunopharma, and Pfizer. Yvonne Efebera has served as a consultant and on advisory boards for Takeda, Oncopeptide, Janssen, GSK, Alnylam, Sanofi, and Pfizer. She has received research support from BMS/Celgene. She has served on independent adjudication committees for Takeda and Orca. Bakri Elsheikh received research funding from Biogen, Genentech, Alexion, Pharnext, and Viela Bio and served as a consultant for Biogen, Genentech, and Argenx.

## Supporting information


Data S1:

## Data Availability

The data that supports the findings of this study are available in the supplementary material of this article.
