# Supplementary material for: Lenalidomide in the treatment of anti‐myelin‐associated glycoprotein neuropathy: A phase 1 study to identify the maximum tolerated dose
Source: Eur J Neurol. 2023 Nov 28;31(3):e16164. doi: 10.1111/ene.16164 (PMC11235859; doi:10.1111/ene.16164)
Supplement: Supplementary file 1 — Data S1: [file ENE-31-e16164-s001.docx]

**Supplementary Items**

Supplementary Table 1 - Definition of DLT event ^a^ and corresponding course of action.

Supplementary Table 2 - Clinicometric and Serologic Outcomes, Source Data.

Supplementary Figure 1 – Select Flow Cytometry Markers

**Supplementary Table 1 – Definition of DLT event and corresponding action plan**

| **DLT** | **Definition** |
| --- | --- |
| Neutropenia | If grade 3 or greater on day 1 of cycle: Hold lenalidomide and monitor CBC weekly; if resolves to ≤ grade 2 within 4 weeks, resume lenalidomide with 5mg dose reduction. |
| Febrile Neutropenia | Hold lenalidomide for the remainder of the cycle. Following completion of cycle 1, consider G-CSF administration until there is adequate count recovery ANC≥1000/µL + absence of fever. If neutropenia resolves ≤ grade 2, resume with lenalidomide at 5 mg dose reduction for subsequent cycles |
| Thrombocytopenia | If grade 3 or greater, hold lenalidomide the remainder of the cycle and until platelets ≥ 50,000/µL. Then resume lenalidomide at 5 mg dose reduction for subsequent cycles Platelet transfusion may be given following completion of cycle 1. Platelet transfusion for severe thrombocytopenia may be administered in cycle 1 at investigator discretion following discussion with Principal Investigators. |
| Anemia | For grade 3 or greater, hold lenalidomide for the remainder of the cycle and until hemoglobin recovers to ≤ grade 2. Red cell transfusion may be given. |
| Fatigue (asthenia, malaise) | For intolerable grade 2 or grade 3-4, hold protocol therapy thought to be probable or definitely related to the reaction until resolves to ≤ grade 1, then resume protocol therapy with dose reduction of implicated protocol therapy by one dose level (5 mg for lenalidomide, or 50% for dexamethasone). |
| Rash | If grade 3 or greater, hold lenalidomide until resolves to ≤ grade 2, then resume protocol therapy. Antihistamine (Benadryl, Claritin, Zyrtec) may be given. If grade > 3 rash recurs, hold therapy until rash resolves to < grade 2, resume with one level dose reduction of agent(s) believed to be responsible for rash. |
| Renal insufficiency | For patients with calculated or measured GFR <30ml/min, lenalidomide should be held until creatinine returns to baseline, then 5 mg dose reduction |
| Venous Thromboembolism | All protocol treatment should be held until the patient is adequately anti-coagulated. Patients with recurrent thrombosis despite anti-coagulation should be removed from protocol therapy. |
| Suspected Pregnancy | Protocol therapy should be held until pregnancy is ruled out. Discontinue all protocol therapy if pregnancy is positive |
| Dyspepsia, gastric or duodenal ulcer, gastritis Grade 1-2 (requiring medical management) | Treat with H2 blockers, sucralfate, or omeprazole. If symptoms persist despite above measures, decrease dexamethasone dose by 50% |
| *Acute pancreatitis* | Hold all study therapy until resolution. Do not resume dexamethasone. Consider one level dose reduction (5 mg lenalidomide, 50% dexamethasone) |
| Edema > Grade 3 (limiting function and unresponsive to therapy or anasarca) | Diuretics as needed, and decrease dexamethasone dose by 50%; if edema persists despite above measures, decrease dose another dose level. Discontinue dexamethasone and do not resume if symptoms persist despite second reduction. |
| Confusion or mood alteration > Grade 2 (interfering with function +/- interfering with activities of daily living) | Hold all study therapy until symptoms resolve. Restart with 50% reduction of dexamethasone. If symptoms persist despite above measures, permanently discontinue dexamethasone. Consider one level dose reduction of lenalidomide if agent is felt to be contributing. |
| Muscle weakness > Grade 2 (symptomatic and  interfering with function +/-  activities of daily living) | Hold all study therapy. Restart with 50% dose reduction of dexamethasone dose. If weakness persists despite above measures decrease dose by another 50%. Discontinue dexamethasone and do not resume if symptoms persist. Consider one level dose reduction of lenalidomide if agent is felt to contributing. |
| Hyperglycemia > Grade 3 or more | Hold dexamethasone. Treat with insulin or oral hypoglycemics as needed. If uncontrolled despite above measures, decrease dose by 50%. |

DLTs are defined as occurring during cycle 1 of drug therapy. Any above event occurring after cycle 1 would be classified as a non-DLT serious adverse event and not impact the MTD calculation.

**Supplementary Table 2 – Clinicometric and Serologic Outcomes, Source Data.**

|  | **Outcomes** | **Baseline** | **Cycle 3** | **Cycle 6** | **Cycle 9** | **Cycle 12** | **Cycle 18** | **Cycle 24** |
| --- | --- | --- | --- | --- | --- | --- | --- | --- |
| Patient 1 | ONLS, units | 5 | 6 |  | 2 | 2 | 4 | 3 |
|  | IRODS (raw, non-percentile), units | 28 | 24 |  | 27 | 23 | 31 | 27 |
|  | FSS, units | 27 | 37 |  | 16 | 45 | 38 | 23 |
|  | EQ5D, units | 6 | 6 |  | 6 | 4 | 4 | 6 |
|  | SARA, units | 15.5 | 16 |  | 17 | 9 | 14 | 8.5 |
|  | MRCSS | 57 | 56 |  | 56 | 57 | 57 | 57 |
|  | Jamar R, lbs | 70 | 67 |  | 60 | 60 | 55 | 55 |
|  | Jamar L, lbs | 52 | 60 |  | 45 | 60 | 60 | 45 |
|  | IgM spike mg/dL | 115 |  |  |  | 85 |  |  |
|  | IgM total mg/dL | 245 |  |  |  | 128 |  |  |
|  | MAG, BTU | 102400 |  |  |  | 102400 |  | 102400 |
| Patient 2 | ONLS, units | 1 | 3 | 2 | 1 | 2 | 2 | 1 |
|  | IRODS (raw, non-percentile), units | 46 | 45 | 47 | 45 | 47 | 45 | 46 |
|  | FSS, units | 12 | 17 | 50 | 57 | 9 | 10 | 12 |
|  | EQ5D, units | 3 | 4 | 3 | 2 | 2 | 2 | 2 |
|  | SARA, units | 9 | 9 | 2 | 12 |  | 7.5 | 7.5 |
|  | MRCSS, units | 60 | 60 | 58 | 60 |  | 60 | 58 |
|  | Jamar R, lbs | 68 | 85 | 80 | 82 | 75 | 75 | 75 |
|  | Jamar L, lbs | 75 | 72 | 80 | 78 | 75 | 80 | 72 |
|  | IgM spike mg/dL | 116 |  |  |  | 110 |  |  |
|  | IgM total mg/dL | 438 |  |  |  | 246 |  |  |
|  | MAG, BTU | 102400 |  | 102400 |  | 102400 |  |  |
| Patient 3 | ONLS, units | 3 | 2 | 2 | 2 | 2 |  |  |
|  | IRODS (raw, non-percentile), units | 32 |  | 37 | 39 | 38 |  |  |
|  | FSS, units | 33 | 38 |  | 27 | 37 |  |  |
|  | EQ5D, units | 4 | 3 | 3 | 3 | 4 |  |  |
|  | SARA, units | 3 | 3 |  | 2 |  |  |  |
|  | MRCSS, units | 60 | 60 |  | 60 |  |  |  |
|  | Jamar R, lbs | 98 | 105 |  | 100 | 110 |  |  |
|  | Jamar L, lbs | 86 | 105 |  | 100 | 95 |  |  |
|  | IgM spike mg/dL | 41 |  |  |  | 19 |  |  |
|  | IgM total mg/dL | 157 |  |  |  | 89 |  |  |
|  | MAG, BTU | 102400 |  | 16680 |  | 12800 |  |  |
| Patient 4 | ONLS, units | 3 | 2 |  | 2 | 2 | 2 |  |
|  | IRODS (raw, non-percentile), units |  | 38 | 42 | 38 | 42 | 43 |  |
|  | FSS, units | 44 | 34 | 36 | 28 | 29 | 36 |  |
|  | EQ5D, units | 4 | 4 | 3 | 4 | 3 | 3 |  |
|  | SARA, units | 7 | 3 |  | 3 | 3.5 | 3.5 |  |
|  | MRCSS, units | 54 | 56 |  | 56 | 57 | 57 |  |
|  | Jamar R, lbs | 96 | 100 |  | 100 | 101 | 95 |  |
|  | Jamar L, lbs | 89 | 90 |  | 90 | 100 | 94 |  |
|  | IgM spike mg/dL | 139 |  |  |  | 182 |  |  |
|  | IgM total mg/dL | 755 |  |  |  | 716 |  |  |
|  | MAG, BTU | 102400 |  |  |  | 102400 |  |  |
|  |  |  |  |  |  |  |  |  |
| **(continued)** | **Outcomes** | **Baseline** | **Cycle 3** | **Cycle 6** | **Cycle 9** | **Cycle 12** | **Cycle 18** | **Cycle 24** |
| Patient 5 | ONLS, units | 6 |  | 7 | 6 |  |  |  |
|  | IRODS (raw, non-percentile), units | 22 | 23 | 16 | 22 |  |  |  |
|  | FSS, units | 54 | 49 | 57 | 48 |  |  |  |
|  | EQ5D, units | 8 | 6 | 5 | 6 |  |  |  |
|  | SARA, units | 11.5 | 10.5 | 14.5 | 11.5 |  |  |  |
|  | MRCSS, units | 51 | 54 | 53 | 53 |  |  |  |
|  | Jamar R, lbs | 25 | 14 | 18 | 16 |  |  |  |
|  | Jamar L, lbs | 45 | 43 | 44 | 45 |  |  |  |
|  | IgM spike | 244 |  |  |  |  |  |  |
|  | IgM total | 600 |  |  |  |  |  |  |
|  | MAG, BTU |  |  | 102400 |  |  |  |  |
| Notes: | Patient had pulmonary embolus in cycle 6, but continued in study until end of cycle 9 off drug for next 3 cycles (self-withdrawal) | | | | | | | |
| Patient 6 | ONLS, units | 2 | 3 | 2 | 2 | 2 |  |  |
|  | IRODS (raw, non-percentile), units | 42 | 38 | 41 | 38 | 36 |  |  |
|  | FSS, units | 17 | 20 | 18 | 18 | 36 |  |  |
|  | EQ5D, units | 5 | 4 | 3 | 3 | 4 |  |  |
|  | SARA, units | 5 | 3.5 | 1 | 0 | 4 |  |  |
|  | MRCSS, units | 60 | 60 | 60 | 60 | 60 |  |  |
|  | Jamar R, lbs | 50 | 52 | 48 | 50 | 45 |  |  |
|  | Jamar L, lbs | 45 | 45 | 46 | 47 | 50 |  |  |
|  | IgM spike | 1009 |  |  |  | 400 |  |  |
|  | IgM total | 1396 |  |  |  | 957 |  |  |
|  | MAG, BTU | 102400 |  | 102400 |  | 102400 |  |  |
| Patient 7 | ONLS, units | 2 | 1 | 2 | 1 | 2 |  |  |
|  | IRODS (raw, non-percentile), units | 45 | 43 | 46 | 46 | 45 |  |  |
|  | FSS, units | 44 | 23 | 33 | 38 | 30 |  |  |
|  | EQ5D, units | 4 | 4 | 2 | 2 | 2 |  |  |
|  | SARA, units | 3.5 | 1 | 1.5 | 2.5 | 2 |  |  |
|  | MRCSS, units | 60 | 60 | 60 | 60 | 60 |  |  |
|  | Jamar R, lbs | 100 | 80 | 80 | 69 | 75 |  |  |
|  | Jamar L, lbs | 85 | 85 | 90 | 79 | 80 |  |  |
|  | IgM spike mg/dL |  |  |  |  |  |  |  |
|  | IgM total mg/dL | 237 |  |  |  | 146 |  |  |
|  | MAG, BTU | 102400 |  | 25600 |  | 102400 |  |  |
| Notes: | Patient had pulmonary embolus and deep vein thrombosis at cycle 3 but continued in study until end of cycle 12 on study drug. | | | | | | | |
| Patient 8 | ONLS, units | 1 | 2 | 2 | 6 |  |  |  |
|  | IRODS (raw, non-percentile), units | 47 | 44 | 41 | 31 |  |  |  |
|  | FSS, units | 45 | 46 | 53 | 52 |  |  |  |
|  | EQ5D, units | 3 | 5 | 5 | 5 |  |  |  |
|  | SARA, units | 2 | 6.5 | 10.5 | 10 |  |  |  |
|  | MRCSS, units | 59 | 60 | 58 | 56 |  |  |  |
|  | Jamar R, lbs | 75 | 80 | 76 | 66 |  |  |  |
|  | Jamar L, lbs | 68 | 94 | 76 | 50 |  |  |  |
|  | IgM spike mg/dL | 525 |  |  |  |  |  |  |
|  | IgM total mg/dL | 1116 |  |  |  |  |  |  |
|  | MAG, BTU | 102400 |  | 102400 |  |  |  |  |
| Notes: | Patient had pulmonary embolus and deep vein thrombosis in cycle 9, and ended participation due to both events and disease progression at end of cycle 9 (self-withdrawal) | | | | | | | |
| **(continued)** | **Outcomes** | **Baseline** | **Cycle 3** | **Cycle 6** | **Cycle 9** | **Cycle 12** | **Cycle 18** | **Cycle 24** |
| Patient 9 | ONLS, units | 4 | 4 |  |  |  |  |  |
|  | IRODS (raw, non-percentile), units | 33 | 38 |  |  |  |  |  |
|  | FSS, units | 44 | 38 |  |  |  |  |  |
|  | EQ5D, units | 2 | 4 |  |  |  |  |  |
|  | SARA, units | 13 | 13 |  |  |  |  |  |
|  | MRCSS, units | 60 | 60 |  |  |  |  |  |
|  | Jamar R, lbs | 20.5 | 45 |  |  |  |  |  |
|  | Jamar L, lbs | 21.5 | 48 |  |  |  |  |  |
|  | IgM spike mg/dL |  |  |  |  |  |  |  |
|  | IgM total mg/dL | 196 |  |  |  |  |  |  |
|  | MAG, BTU | 51200 |  |  |  |  |  |  |
| *Notes:* | Patient was electively withdrawn by study coordinators at end of cycle 3 due to noncompliance with study drug schedule (overdosing) and risk of untoward adverse events | | | | | | | |
| Patient 10 | ONLS, units | 2 |  |  |  |  |  |  |
|  | IRODS (raw, non-percentile), units |  |  |  |  |  |  |  |
|  | FSS, units |  |  |  |  |  |  |  |
|  | EQ5D, units |  |  |  |  |  |  |  |
|  | SARA, units |  |  |  |  |  |  |  |
|  | MRCSS, units | 58 | 60 |  |  |  |  |  |
|  | Jamar R, lbs |  |  |  |  |  |  |  |
|  | Jamar L, lbs |  |  |  |  |  |  |  |
|  | IgM spike mg/dL | 100 |  |  |  |  |  |  |
|  | IgM total mg/dL | 315 |  |  |  |  |  |  |
|  | MAG, BTU | 102400 |  |  |  |  |  |  |
| Notes: | Patient experienced febrile neutropenia, pneumonia, and drug-rash during cycle 2, prompting hospitalization, and for which he ended participation in study (self-withdrawal). Safety visit was conducted at end of cycle 3 with partial outcome data collected. | | | | | | | |
| Patient 11 | ONLS, units | 6 | 7 | 6 | 6 | 4 |  |  |
|  | IRODS (raw, non-percentile) | 22 | 28 | 29 | 30 | 29 |  |  |
|  | FSS, units | 22 | 28 | 23 | 26 | 15 |  |  |
|  | EQ5D, units | 5 | 7 | 6 | 6 | 4 |  |  |
|  | SARA, units | 27.5 | 19 | 18.5 | 19.5 | 15.5 |  |  |
|  | MRCSS, units | 53 | 51 | 53 | 55 | 53 |  |  |
|  | Jamar R, lbs | 58 | 65 | 60 | 62 | 62.5 |  |  |
|  | Jamar L, lbs | 44 | 37 | 40 | 40 | 50 |  |  |
|  | IgM spike, mg/dL | 200 |  |  |  | 100 |  |  |
|  | IgM total mg/dL | 659 |  |  |  | 435 |  |  |
|  | MAG, BTU | 102400 |  |  |  | 51303 |  |  |

Abbreviations: ONLS = overall neuropathy limitations scale; IRODS = inflammatory Rasch-built overall disability scale; FSS = Fatigue severity scale; EQ-5D = EuroQol-5 Dimension; SARA = Scale for the Assessment and Rating of Ataxia; MRCSS = Medical Research Council Summated Score; MAG = myelin associated glycoprotein titer; BTU = Buhlmann Titer Units.

**Supplementary FIgure 1 – Select Flow Cytometry Markers**


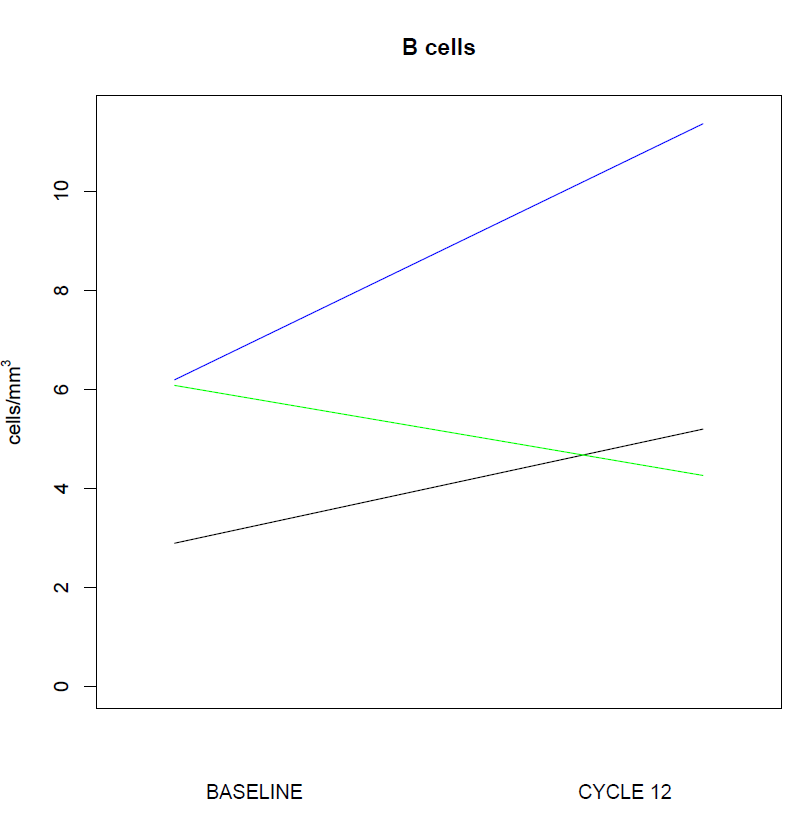

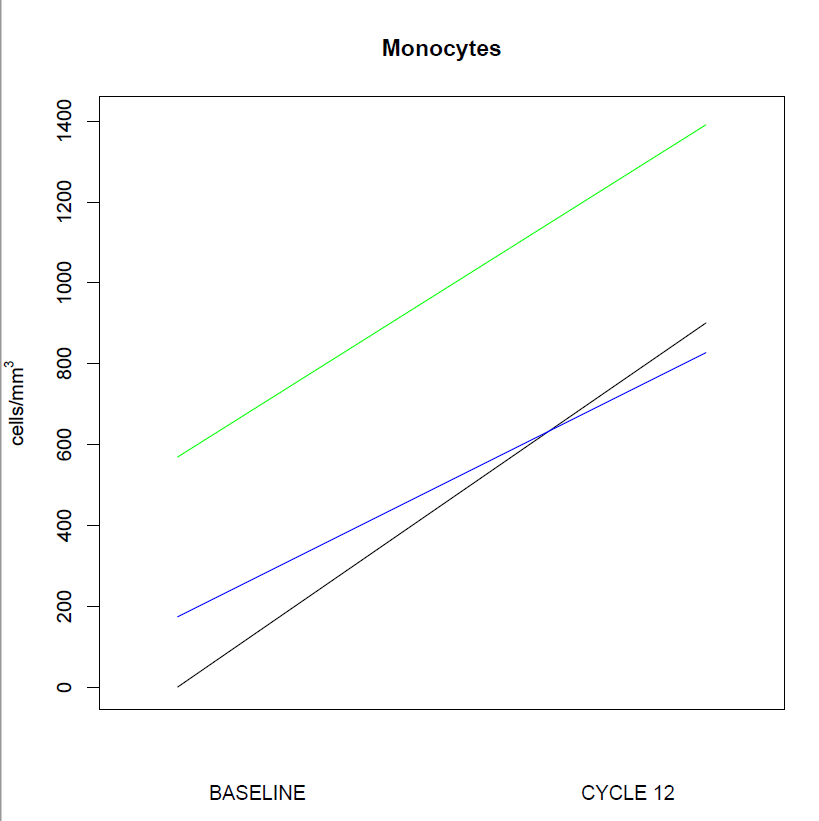


Flow cytometry measures were conducted on 3 patients at baseline and cycle 12 into treatment. All 3 subjects were from the Ohio State University. *Black line* = Subject 2 (clinical responder); *Blue line* = Subject 2 (clinical non-responder), *Green line =* Subject 4 (clinical responder). Clinical response defined as an improvement of > 1 on the Overall Neuropathy Limitations Scale.
